# Complete Genome Sequences of Igbo-Ora and Babanki Alphavirus Strains Isolated in the Central African Republic in the 1960s and 1970s

**DOI:** 10.1128/MRA.00868-19

**Published:** 2019-10-10

**Authors:** Vianney Tricou, Benjamin Selekon, Ousmane Faye, Antoine Gessain, Mirdad Kazanji, Emmanuel Nakouné, Nicolas Berthet

**Affiliations:** a Institut Pasteur de Bangui, Bangui, Central African Republic; b Institut Pasteur de Dakar, Dakar, Senegal; c Institut Pasteur, Epidemiology and Physiopathology of Oncogenic Viruses, CNRS UMR3569, Paris, France; d Institut Pasteur, Unité Environnement et Risques Infectieux, Cellule d’Intervention Biologique d’Urgence, Paris, France; Portland State University

## Abstract

Vector-borne viruses are becoming increasingly important from a public health standpoint with the emergence or reemergence of viruses and extension of the areas at risk. Here, we report the whole-genome sequences of two alphaviruses, namely, one Igbo-Ora virus and one Babanki virus, that were isolated several decades ago in Africa from human serum.

## ANNOUNCEMENT

Igbo-Ora and Babanki viruses belong to the Alphavirus genus and Togaviridae family and cause diseases in humans. Both of them are arthropod-borne viruses. Igbo-Ora virus was first isolated in Nigeria (1). Initially considered a separate alphavirus species, Igbo-Ora virus was later confirmed to be a strain of O’nyong-nyong virus (2). Babanki virus was first isolated in Northwest Cameroon in 1969 and is a strain of Sindbis virus (3).

Here, we report the whole-genome sequences of HB67652 and HB741570 viruses (an Igbo-Ora virus and a Babanki virus), which were both isolated from human serum specimens collected in the Central African Republic in 1966 and 1975, respectively. These viruses were isolated and amplified by serial passages in brains of newborn mice (unknown passage numbers). Brains were homogenized in Hanks’ solution and centrifuged. Supernatants were lyophilized and stored in sealed glass vials at room temperature until 2012. Viral genomic material was extracted from lyophilizates that were resuspended in phosphate-buffered saline using a QIAamp viral RNA minikit (Qiagen). The extracted RNA was retrotranscribed into cDNA using SuperScript III enzyme and random hexamers (Life Technologies). The cDNA fragments were ligated and then amplified using the phi29 DNA polymerase and random hexamers (4). Amplified DNA was fragmented using an M220 ultrasonicator (Covaris) and was used to construct genomic libraries with a NEBNext Ultra DNA library prep kit according to the manufacturer’s recommendations (New England Biolabs). Sequencing was performed using a HiSeq 2000 sequencer (Illumina) (5). More than 14 and 20 million 100-bp paired-ended reads were generated for HB741570 and HB67652, respectively. The quality of initial reads was assessed by FastQC (6). Because of the chimeric reads generated by Phi29 DNA polymerase, which prevent *de novo* genome assembly, a similarity-based approach using BLASTN was applied to eliminate nonviral chimeric parts of the reads. GenBank accession numbers HM147984 and AF079457 (which were the only 2 complete genomes available) were used as targeted sequences. Finally, 697,876 and 1,412,595 viral sequences, covering overall the target sequences from their 5′ to 3′ ends, were obtained and used for whole-genome assembly with SPAdes version 3.1.0 (7, 8).

For HB67652 and HB741570, the lengths of the whole-genome sequences were 11,818 and 11,714 nucleotides, with average coverages of 3,221× and 1,933× and GC contents of 48.1% and 51.6, respectively. The coding sequence lengths of HB67652 were 7,542 and 3,744 nucleotides for the 1st and 2nd open reading frame (ORF), respectively. The coding sequence lengths of HB741570 were 7,548 and 3,738 nucleotides for the 1st and 2nd ORF, respectively. Of note, a leaky stop codon near the nsP3 gene 3′ end is present in HB741570, unlike in HB67652. HB67652 and HB741570 share >99% nucleic acid identity with the only 2 other complete genomes of Igbo-Ora and Babanki viruses currently available in GenBank (accession numbers AF079457 and HM147984, respectively).

While Igbo-Ora virus and Sindbis viruses have confirmed potential to cause widespread epidemics in humans, the epidemic potential of Babanki virus remains unclear (9, 10). Further analysis of these whole-genome sequences may improve understanding of the transmission cycles and the complex dynamics that underlie the epidemiology of these pathogens.

### Data availability.

These whole-genome sequences are available in GenBank under accession numbers MF409176 and MF409178. The raw sequencing data have been submitted to the SRA database under the accession numbers SRR9712540 and SRR9712544.

## References

[1] Moore DL, Causey OR, Carey DE, Reddy S, Cooke AR, Akinkugbe FM, David-West TS, Kemp GE 1975 Arthropod-borne viral infections of man in Nigeria, 1964–1970. Ann Trop Med Parasitol 69:49–64. doi:10.1080/00034983.1975.11686983. 1124969

[2] Lanciotti RS, Ludwig ML, Rwaguma EB, Lutwama JJ, Kram TM, Karabatsos N, Cropp BC, Miller BR 1998 Emergence of epidemic O’nyong-nyong fever in Uganda after a 35-year absence: genetic characterization of the virus. Virology 252:258–268. doi:10.1006/viro.1998.9437. 9875334

[3] Karabatsos N 1985 International catalogue of arboviruses, including certain other viruses of vertebrates, 3rd ed American Society of Tropical Medicine and Hygiene for the Subcommittee on Information Exchange of the American Committee on Arthropod-Borne Viruses, San Antonio, TX.

[4] Berthet N, Reinhardt AK, Leclercq I, van Ooyen S, Batéjat C, Dickinson P, Stamboliyska R, Old IG, Kong KA, Dacheux L, Bourhy H, Kennedy GC, Korfhage C, Cole ST, Manuguerra JC 2008 Phi29 polymerase based random amplification of viral RNA as an alternative to random RT-PCR. BMC Mol Biol 9:77. doi:10.1186/1471-2199-9-77. 18771595 PMC2535778

[5] Tricou V, Berthet N, Descorps-Declere S, Nakouné E, Kazanji M 2014 Complete genome sequences of two Middelburg viruses isolated from arthropods in the Central African Republic. Genome Announc 2:e01078-14. doi:10.1128/genomeA.01078-14. 25342688 PMC4208332

[6] Andrews S 2010 FastQC: a quality control tool for high throughput sequence data. http://www.bioinformatics.babraham.ac.uk/projects/fastqc.

[7] Bankevich A, Nurk S, Antipov D, Gurevich AA, Dvorkin M, Kulikov AS, Lesin VM, Nikolenko SI, Pham S, Prjibelski AD, Pyshkin AV, Sirotkin AV, Vyahhi N, Tesler G, Alekseyev MA, Pevzner PA 2012 SPAdes: a new genome assembly algorithm and its applications to single-cell sequencing. J Comput Biol 19:455–477. doi:10.1089/cmb.2012.0021. 22506599 PMC3342519

[8] Berthet N, Descorps-Declère S, Nkili-Meyong AA, Nakouné E, Gessain A, Manuguerra JC, Kazanji M 2016 Improved assembly procedure of viral RNA genomes amplified with Phi29 polymerase from new generation sequencing data. Biol Res 49:39. doi:10.1186/s40659-016-0099-y. 27605096 PMC5015205

[9] Lhuillier M, Cunin P, Mazzariol MJ, Monteny N, Cordellier R, Bouchite B 1988 A rural epidemic of IgBo Ora virus with interhuman transmission in the Ivory Coast. Bull Soc Pathol Exot Filiales 81:386–395. 2846194

[10] Braack L, Gouveia De Almeida AP, Cornel AJ, Swanepoel R, de Jager C 2018 Mosquito-borne arboviruses of African origin: review of key viruses and vectors. Parasit Vectors 11:29. doi:10.1186/s13071-017-2559-9. 29316963 PMC5759361

